# An updated perspective on immunoglobulin replacement in chronic lymphocytic leukaemia in the era of targeted therapies

**DOI:** 10.3389/fonc.2023.1135812

**Published:** 2023-04-06

**Authors:** Sujoy Khan, David Allsup, Stefano Molica

**Affiliations:** ^1^ Department of Immunology and Allergy, Castle Hill Hospital, Hull University Teaching Hospital National Health Service (NHS) Trust, Cottingham, United Kingdom; ^2^ Department of Haematology, Castle Hill Hospital, Hull University Teaching Hospital NHS Trust, Cottingham, United Kingdom; ^3^ Centre for Biomedicine, Hull York Medical School, University of Hull, Hull, United Kingdom

**Keywords:** CLL, hypogammaglobulinemia, Ig replacement, immunomodulation, restoration immunity

## Abstract

Chronic lymphocytic leukaemia (CLL) is a malignancy of clonally expanded antigen-switched, neoplastic, mature B cells. CLL is characterised by a variable degree of immunosuppression and secondary hypogammaglobulinemia. B-cell depleting therapies have historically been deployed with a proportion of patients becoming resistant to multiple lines of treatment with an associated worsening of immunosuppression and heightened infection risk. Advances in molecular diagnostics and the development of new therapies targeting Bruton’s tyrosine kinase and B-cell lymphoma-2 have resulted in novel insights into the cellular mechanisms associated with an increased infection risk and T-cell escape from the complex tumour environment found in CLL. Generally, immunoglobulin replacement therapy with polyvalent human immunoglobulin G (IgG) is indicated in patients with recurrent severe bacterial infections and low IgG levels, but there is no consensus on the threshold IgG level for initiation of such therapy. A proportion of CLL patients have residual IgG production, with preserved quality of the immunoglobulin molecules, and therefore a definition of ‘IgG quality’ may allow for lower dosing or less frequent treatment with immunoglobulin therapy in such patients. Immunoglobulin therapy can restore innate immunity and in conjunction with CLL targeted therapies may allow T-cell antigen priming, restore T-cell function thereby providing an escape from tumour-associated autoimmunity and the development of an immune-mediated anti-tumour effect. This review aims to discuss the mechanisms by which CLL-targeted therapy may exert a synergistic therapeutic effect with immunoglobulin replacement therapy both in terms of reducing tumour bulk and restoration of immune function.

## Introduction

Chronic lymphocytic leukaemia (CLL) is the most common form of leukaemia in the Western World, with around 4700 new cases diagnosed each year in the United Kingdom (1). Current guidelines suggest that treatment for CLL is indicated only for symptomatic or rapidly progressive disease (2). Therefore, the current standard of care for patients with early CLL is a ‘watch-and-wait’ approach that includes regular physical examination and laboratory testing to assess disease status over time. This approach is based upon research which has demonstrated that pre-emptive therapy for asymptomatic CLL has no effect on overall survival, even when novel targeted agents are utilised, as shown in the recently concluded CLL12 trial (3). The treatment landscape for symptomatic CLL has rapidly evolved with the development of multiple targeted agents that have improved survival outcomes. However, despite these improvements in treatment, CLL remains incurable with infection being the leading direct cause of death (4).

The inherent disease-related immune dysfunction along with secondary hypogammaglobulinemia, decreased cell-mediated immunity (T-cell dysfunction), and immunosuppression related to B-cell directed therapies are the major causes of the increased susceptibility to infection in CLL. Finally, although the mechanism of CLL-associated hypogammaglobulinemia is largely unknown, factors such as T-cell dysfunction and the abnormal cytokine environment (such as increased tumour necrosis factor–alpha [TNF-α] level) may affect immunoglobulin production (5).

Current guidelines recommend immunoglobulin replacement therapy (IgRT) to reduce the risk of bacterial infections and hospitalization in patients with recurrent severe infections and low immunoglobulin G (IgG) levels (6). In addition, there is evidence that IgRT with polyvalent human IgG has immunomodulatory effects on the innate immune system, whilst the effect of such therapy on B-cell function appears to be more complex and poorly defined (7).

IgRT also exerts effects upon other important immune cells, with a resultant increase in CD4+ T-cell numbers, a reduction in exhaustion markers on both CD4+ and CD8+ T-cells, a transient rise in regulatory T-cells (Tregs) along with effects on invariant-natural killer T-cells (iNKT) and alterations in serum IL-2 and TNF-α (7, 8). Whether such changes are IgRT-induced immunomodulatory alterations that may translate into a change in the cytokine-mediated immunosuppressive tumour microenvironment (TME) of CLL is debated. In fact, subcutaneously administered immunoglobulin therapy (scIgRT) at high doses decreased CD83 expression with consequential inhibition of B-cell receptor (BCR) signalling and reduced TNF-α production with *in-vitro* studies showing an associated decrease in the survival of CLL cells (9). Although such *in-vitro* findings may not precisely reflect responses to high-dose immunoglobulin therapy *in vivo*, these results set the stage for a more comprehensive assessment of IgRT activity in CLL.

## Disruption and chaos of the immune system in persons with CLL

It is a general characteristic of malignancies that the cancer cell manipulates the immune system to the advantage of the tumour, and CLL is no different in this respect.

CLL almost uniformly evolves from the precursor condition monoclonal B-lymphocytosis (MBL) characterized by the co-expression of CLL-like surface markers (CD19, CD5, and CD23, weak expression of CD20 and CD79b) with less than 5 x 10^9^/L B-cells in the circulation. Progression of MBL to CLL is characterized by continuous clonal evolution with the acquisition of high-risk genetic driver mutations that may lead to symptomatic disease (10–12).

**Figure 1:**
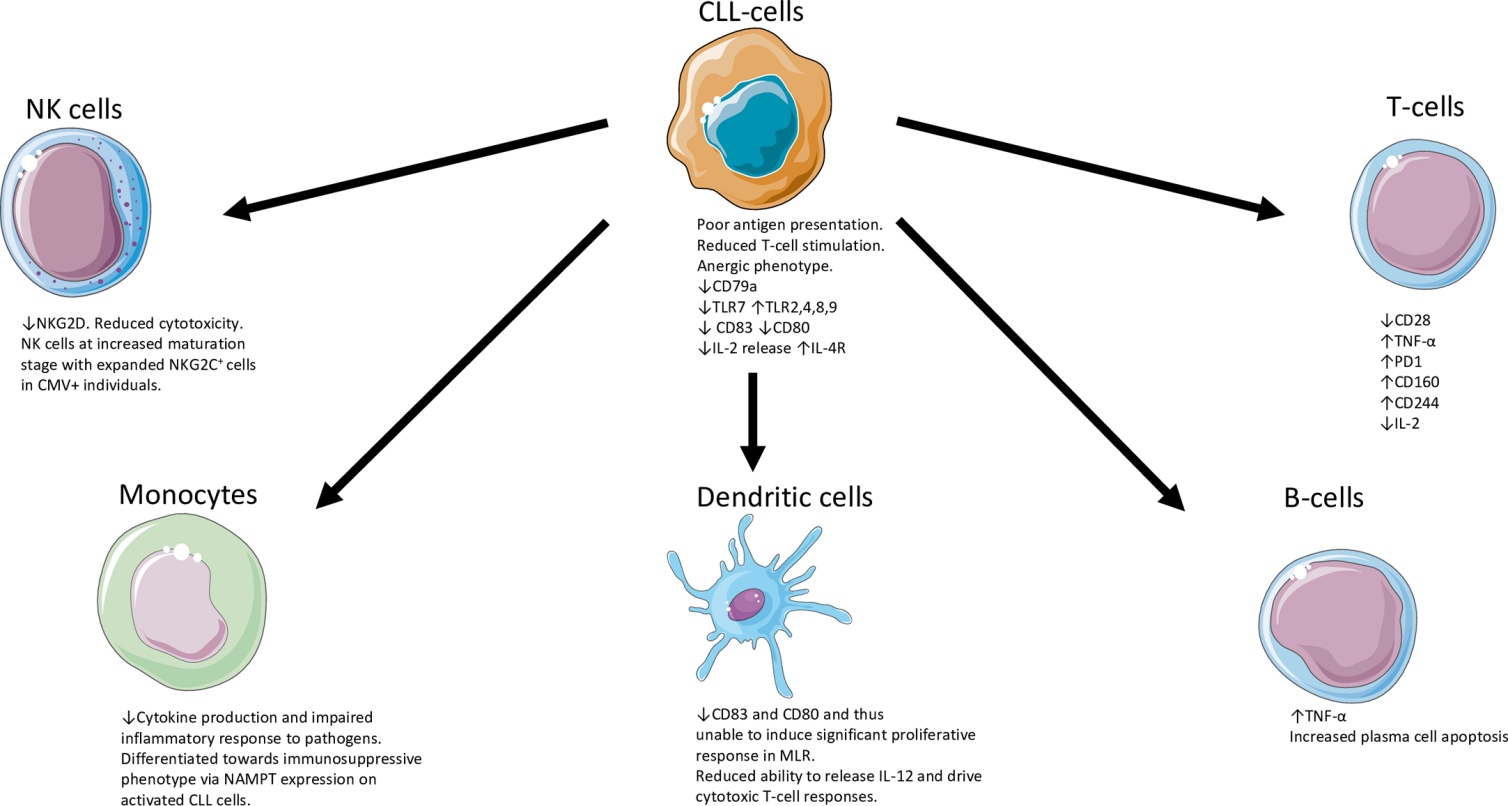
Overview of the disruption and chaos of the immune system in patients with CLL (5, 15–29). CLL, chronic lymphocytic leukaemia; IL-2, interleukin 2; IL-4R, interleukin-4 receptor; NK, natural killer; TLR, Toll-like receptor; MLR, mixed lymphocyte reaction; NAMPT, nicotinamide phosphoribosyltransferase; PD1, Programmed death protein 1; TNF, Tumour necrosis factor; TLR, Toll receptor. The Figure was partly generated using Servier Medical Art, provided by Servier, licensed under a Creative Commons Attribution 3.0 unsupported license.

The skewed BCR repertoire found in CLL is illustrated by the utilisation of specific immunoglobulin heavy-chain gene rearrangements found in *IGHV* 4-34, 3-23, 1-69 as well the expression of highly stereotypic BCRs (13). Similarly, the expression of zeta-associated protein 70 (ZAP70) or CD38, deletions of chromosome 11q or 17p, unmutated immunoglobulin heavy chain (*IGHV*) genes indicative of pre-germinal centre CD5+ B-cells, mutations in *TP53*, Ataxia-Telangestasia Mutated (*ATM), DDX3X, NOTCH1*, spicing factor 3b subunit 1 (*SF3B1)* genes are all associated with high-risk disease. In contrast, deletions of chromosome 13q and highly mutated *IGHV* genes (indicative of post-germinal centre CD5+ B-cells) are associated with a favourable prognosis (14). Although to a lesser extent when compared to CLL, high-count MBL can carry CLL-specific genomic aberrations with associated altered T-cell function (10). We have tried to provide an overview of the disruption and chaos of the immune system in patients with CLL (**Figure 1**) evident at multiple levels of the cellular immune response.

The nodal TME provides essential survival signals whereby CLL-cell-secreted soluble factors interact with T-cells and tumour-infiltrating macrophages leading to a tumour supportive M2 phenotype. M2-macrophages upregulate PD-1 expression on T-cells to induce suppression of cytotoxic T-cells and T-cell activation whilst promoting Treg differentiation (15). IL-4 present within the TME upregulates CLL-cell expression of surface IgM (sIgM), CD79b and associated BCR expression that rescues anergic B-CLL cells from signal incompetency as they enter lymph nodes (16). These processes finally shape the immunosuppressive features typical of CLL with the consequential development of hypogammaglobulinemia. Autoimmune haemolytic anaemia and thrombocytopenia are common consequences of such CLL-associated immune dysregulation (5).

## Prevalence and impact of hypogammaglobulinaemia in CLL

The most common and clinically relevant impact of CLL on the affected persons immunological status is hypogammaglobulinaemia (decreased serum immunoglobulin levels of all three isotypes IgG, IgA, and IgM). Such a reduction in immunoglobulins is present in up to a third of persons with CLL at diagnosis, and a further third develop hypogammaglobulinemia as the disease progresses, or because of administered therapy (5). Of note, a proportion of CLL patients retain a degree of residual IgG production, with preserved quality of the immunoglobulin molecules. This residual IgG production may have implications for the ability to mount an immunological response to vaccination in persons with CLL. Generally, the primary immune response to a novel antigen is suppressed in persons with CLL compared to the brisk anamnestic immune response in healthy persons (30). A recent prospective non-interventional trial suggests that multiple doses of a COVID19 vaccine can result in high rates of seroconversion in CLL (94.2%) and MBL (100%) (31). Whether the repeated immune stimulus or a post-treatment recovering immune status is driving the response (or if this is sustained) is unclear and needs further investigation.

The evolution of hypogammaglobulinaemia parallels disease progression with data showing faster rates of decline in immunoglobulin levels in those patients who required treatment (9, 32). Parikh et al. showed that patients with advanced stage CLL were more likely to have hypogammaglobulinemia at diagnosis, and the median time to first treatment (TTFT) was shorter compared to patients with normal IgG (3.8 years *vs* 7.4 years) (32). Spaner and colleagues showed that patients whose IgG declined at a rate of 0.85 ± 0.14 g/L/year were those who required treatment within 4.5 ± 0.4 years from initial diagnosis (n = 51) versus a slower rate of 0.27 ± 0.04 g/L/year in those remained untreated after 8.5 ± 0.5 years (n = 40) (9). Of note in the same study, patients with indolent disease (n = 25) maintained endogenous IgG levels above 8 g/L. Future studies should clarify whether modification of the dose schedule of IgRT to achieve higher than conventional IgG target levels may impact on the natural history of CLL.

Although CLL represents a malignant proliferation of B-lymphocytes, the proliferating clonal B-cells retain the ability to secrete immunoglobulin (33). Li et al. showed that non-clonal B-cells derived from persons with CLL and CLL-cells have enhanced surface and serum expression of FcμR, and serum FcμR levels correlated significantly with circulating lymphocyte numbers but not with *IGHV* mutation status or Rai stage (34). This observation may partly explain why serum immunoglobulin levels do not correlate with survival in CLL patients including those treated with Bruton’s tyrosine kinase-inhibitors (BTKi).

In an analysis of 1113 cases with Binet Stage A CLL, low IgA level was associated with a shortened TTFT but neither baseline hypogammaglobulinemia nor the presence of a paraprotein influenced overall survival. However, in this cohort lymphadenopathy, serum β2-microglobulin, CD38, and ZAP70 expression all had prognostic significance (35). A Chinese study of CLL patients with and without a paraprotein found that those with a paraprotein had significantly shorter survival times compared to those without a paraprotein. Factors related to the presence of a paraprotein were advanced Binet stage, autoimmune haemolytic anaemia, elevated serum β2-microglobulin, elevated thymidine kinase, unmutated *IGHV*, expression of ZAP-70 or CD38-positive and cytogenetic abnormalities such as del(17p13) or del(11q22.3) (36). It is entirely possible that the presence of a paraprotein in CLL may indicate a higher disease burden. Such observations in relation to the presence of a paraprotein and disease burden have also been made in Waldenstrom’s Macroglobulinaemia where autoimmunity associated with a loss of immune tolerance permits the emergence of B-cell clones characterised by a paraprotein which can directly contribute to morbidity (37). In addition, a study by Mozas and colleagues reported 30% prevalence of serum monoclonal components in persons with CLL in a single centre. Such patients with a monoclonal component had shortened survival, irrespective of age, with biclonal paraproteins associated with the shortest survival outcomes (38).

## IgRT in the era of targeted agents: More evidence is needed

It is generally agreed that persons with CLL with significant hypogammaglobulinemia characterized by an IgG of less than 4g/L, in the presence of recurrent or severe bacterial infection despite antibiotic prophylaxis, are candidates for long-term IgRT (6). IgRT is usually administered at replacement doses of 0.2-0.4g/kg per month (39). The immunoglobulin trough level, monitored whilst on replacement therapy, can be used to assess the effectiveness of IgRT and could help to modify subsequent dosing of IgRT. Measurement of immunoglobulin levels at different times to evaluate the peak and trough levels should help to monitor the response to IgRT. However, the clinical response remains the most important parameter on which to base efficacy assessments of IgRT and close clinical assessment of the nature and frequency of infections should be undertaken whilst on such therapy.

Whilst prophylaxis with IgRT was effective in an early study of patients with recurrent severe infections and a serum IgG<3g/L, a crossover study found that restoration of serum IgG levels did not parallel a decrease in the number of severe infectious episodes (40, 41). This could therefore mean that the ‘quality’ of IgG is more important than the level measured (‘nephelometric quantity’) and that absent functional or specific anti-microbial antibodies could be an important consideration for prophylactic IgRT (42). Of note, a feasibility study showed a small difference in efficacy between prophylactic IgRT and prophylactic oral antibiotics but there was no significant difference in time to first major infection between the two treatment arms (43).

A systematic review and meta-analysis of randomised-controlled trials of immunoglobulin prophylaxis concluded that although IgRT use in lymphoproliferative disorders and plasma cell dyscrasias does not provide a survival benefit such therapy does prevent major infections with decreased numbers of infections (44). However, there is inter-trial heterogeneity with respect to patient selection and such studies are insufficiently powered for mortality outcomes (**Table 1**). Finally, the evidence with respect to IgRT in CLL mainly relies on studies that pre-date the modern chemoimmunotherapies and targeted agents currently utilised for the treatment of CLL.

**Table 1:** Randomised clinical trials on effect of immunoglobulin replacement therapy in CLL patients with hypogammaglobulinemia (40, 43, 45–48).

First author / country/ year published	Numbers of patients, single or multicentre, eligibility criteria	Intervention	Findings
Cooperative CLL UK 1988	n=84, multicentre Serum IgG<50% of lower limit of normal, or history of serious infections, or both	IVIG Gammagard 400mg/kg vs placebo (0.9% sodium chloride) every 4 weeks for 12 months	Patients on IgRT had significantly fewer bacterial infections than those on placebo (23 vs. 42; P = 0.01) No significant difference in the incidence of nonbacterial infection
Griffiths / UK / 1989 (study period 1984-1987)	n=12 (CLL, n=8), single centre Serum IgG<3.5 g/L and ≥1 serious infection	Randomized, double-blind, crossover study of IVIG Gammagard 400mg/kg vs placebo (0.9% sodium chloride) every 3 weeks for 12 months	50% on IVIG were infection-free; serum IgG >6.4 g/L correlated with reduced bacterial infections
Boughton/ UK / 1995	n=42, multicentre Serum IgG<5.5 g/L and ≥2 infections in the last year	Prophylactic IVIG (Sandoglobulin 18g) vs placebo (0.6g albumin) every 3 weeks for 1 year Patients on placebo commenced on IgRT if >3 infections, and those on IgRT dose increased to 24g	10 patients with serum IgG<3g/L experienced 65% of infections (total 122 episodes) with rapid rise in IgG on IgRT Of those patients commenced on higher dose, 50% remained infection-free
Molica/ Italy / 1996	n=42, single centre Serum IgG<6 g/L and ≥1 serious infection in the preceding 6 months	Randomized, crossover IVIG 0.3g/kg (Vena-N) every 4 weeks vs no treatment for 6 months, then switched to observation or IVIG for 1 year; then IVIG or no therapy for 6 months	Significantly lower infectious episodes was observed during IVIG prophylaxis (n=30), same applied to the 17 patients who completed 12 months of either observation or IVIG prophylaxis
Mustafa/NY, USA/2021 (2019–2020)	n=9, single centre Criteria: Serum IgG ≥4 g/L and decreased vaccine responses against tetanus, diphtheria and pneumococcus (infection history not a criterion)	9 patients completed 24 weeks of ScIg (Hizentra 20% at 0.13g/kg/week once weekly)	One patient discontinued therapy due to fatigue IgG levels increased significantly within 4 weeks, with decreased reliance on antibiotics (non-neutropenic infections), but declined three months after stopping therapy
McQuilten / Australia & New Zealand / 2021 (RCT 2017-2020)	n=60 (CLL, n=29), multicentre Hypogammaglobulinemia due to haematological malignancy, serum IgG <4g/L (without paraprotein), severe bacterial infections	Phase II multicentre feasibility study IgRT (IVIG 0.4g/kg every 4 weeks, or ScIg 0.1g/kg once every week) vs oral antibiotics (TMP-SMX 160/800mg daily) for 1 year (1:2 ratio)	74% patients in the Ig arm and 64% in the antibiotic arm were free of infection at 12 months 76% (95%CI 53-92) in Ig arm and 71% (95% CI 55-84) in antibiotic arm alive at 12 months
Visentin/Italy/ Retrospective (period of patients’ enrollment not available)	CLL, n= 116 Multicenter Hypogammaglobulinemia and recurrent infections according to the Italian drug agency (AIFA) indications. 25% on therapy with ibrutinib.	Retrospective multicenter 49 patients received IVIG 88 SCIG	Patients receiving SCIG achieved higher IgG after at least +6 months. SCIG decreased the cumulative incidence of first (HR 0.39 p < 0.0001) and second (HR 0.56 p = 0.0411) infection more than IVIG.

CLL, chronic lymphocytic leukaemia; IgG, immunoglobulin G; IgRT, immunoglobulin replacement therapy; IVIG, intravenous immunoglobulin; ScIG, subcutaneous immunoglobulin.

During the past decade, the utilisation of BTKi for CLL treatment has grown rapidly, radically changing the treatment landscape of this disease. Both innate and adaptive immunity may be impacted by BTKi treatment. Ibrutinib, the first-in-class BTKi, induces a partial restoration of normal B-cell numbers and humoral immunity during the first two years of therapy. Improvements in serum IgA in BTKi-treated persons were inversely correlated with the infection rate. Acalabrutinib is a newer, more selective BTKi; however, the effect of this agent on humoral and cellular immunity is similar to that observed with ibrutinib and translates into a partial humoral immune reconstitution with a decreased risk of infection, although a degree of immunodeficiency likely persists.

These data pertaining to BTKi-induced immune reconstitution suggests that a reassessment of immunoprophylaxis with IgRT is needed in the era of targeted agents with regards to the timing of discontinuation of IgRT after immune reconstitution is achieved. Studies of infection and immune reconstitution in BTKi-treated persons should also employ uniform definitions of infection severity, and patient-reported outcomes.

## Maximazing the efficacy of IgRT in CLL: Early initiation and subcutaneous preparations

**Figure 2:**
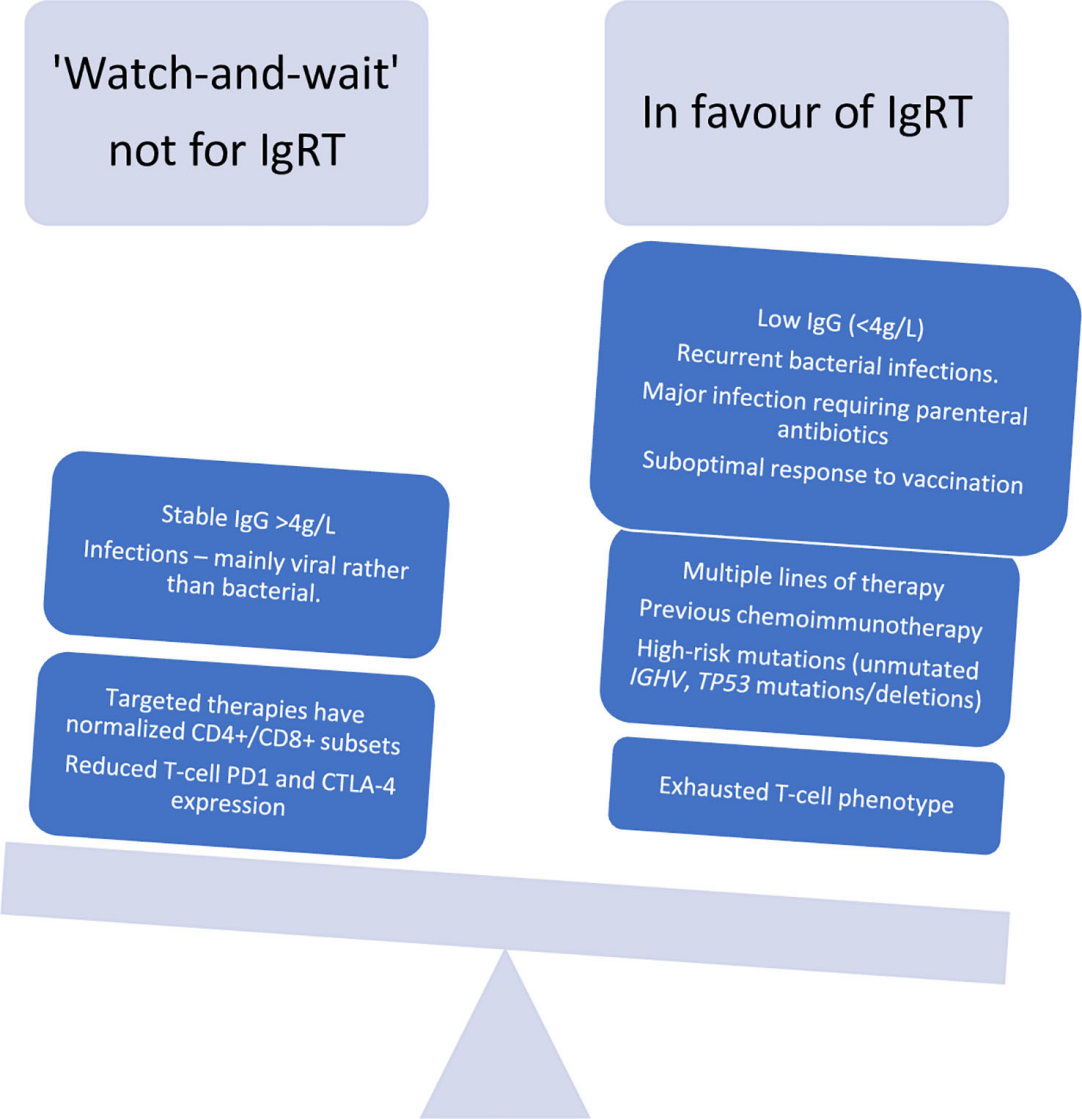
A re-look at indications for immunoglobulin replacement therapy in CLL in the era of targeted therapies.

The prevailing paradigm underpinning current guidelines of IgRT usage in CLL is that such an approach should be deployed in those patients with reduced serum IgG levels and one major infection. However, the first major infectious episode could be fatal, especially in certain patients. This implies that a pre-emptive treatment could be offered to selected patients. In **Figure 2** we weigh the arguments for, and against, an ‘earlier’ or “pre-emptive” IgRT treatment in CLL patients. Apart from symptomatic severe hypogammaglbulinemia, other patient- or CLL-related factors could favour consideration of earlier initiation of prophylactic IgRT. Such patient-specific factors could include the ‘quality’ of low IgG as assessed by a poor vaccine responses (<2.3 x fold response to vaccination), multiple prior lines of therapy, high-risk CLL genomic changes (unmutated *IGHV* genes, adverse cytogenetic features or a complex karyotype), or the presence of exhausted T-cell phenotype. Some of these are *in-vitro* findings and require specialised laboratories so therefore may not be routinely available. Prospective studies comparing an earlier versus delayed IgRT usage should clarify the optimal timing of the initiation of IgRT therapy in CLL.

It has been shown that scIgRT is safe, efficacious, and well tolerated when compared to intravenous IgRT (ivIgRT), providing patients with primary or secondary immunodeficiency disorders with an alternative route of Ig administration (49, 50). Some of the key benefits of scIgRT include: no requirement for venous access, the option to deliver therapy at a time and location of the persons choosing as little medical expertise is needed for administration (infusion pump required but generally very simple to use, after adequate training). scIgRT is typically administered more frequently (such as biweekly, weekly, and even daily based on patient need), resulting in steady state concentrations with fewer fluctuations in IgG plasma levels. More recent scIgRT products utilise a vial of human normal immunoglobulin (Immune Globulin 10%) and one vial of recombinant human hyaluronidase (rHuPH20) that increases the permeability of the subcutaneous tissue by temporarily depolymerizing hyaluronan, allowing larger volume of infusions and treatment intervals to be gradually prolonged from once weekly to once every 3 or 4 weeks. The route of administration also plays a major role in the types of adverse events (AEs) observed in persons receiving IgRT therapy, with systemic AEs associated with intravenous administration and local reactions more commonly seen with subcutaneous administration. Our experience with scIgRT in elderly persons with significant co-morbidities has been positive, but ivIgRT is also effective if there is also clinical supervision to monitor for side-effects associated with increased fluid volumes and rates of infusion. Of note, one meta-analysis determined that the systemic AE rate for scIgRT was 0.43%. A recent retrospective study compared the efficacy of subcutaneous immunoglobulin with an intravenous formulation in patients with CLL and secondary antibody deficiency and found that scIgRT results in higher IgG levels and a decreased rate of infections particularly when IgG levels of 6 g/L is reached (51).

Although limited to few retrospective analyses, the results of efficacy and safety analyses suggest that scIgRT should be preferred in clinical practice for persons with CLL and symptomatic hypogammaglobulinaemia.

## Improving the cost-effectiveness of IgRT

The financial cost of IgRT is significant due to high procurement costs and indefinite usage. Unnecessary and indiscriminate use of IgRT could result in product shortages, adverse outcomes and higher healthcare costs. Keegan and colleagues reported on the demand, supply, and patterns of utilisation of IgRT in CLL in Australia and revealed a sustained increased usage of 5.5% per annum but with significant regional variation across geographical areas (52).

Numerous other studies have demonstrated that scIgRT compared to ivIgRT results in reduced resource use and is therefore more cost effective. A study comparing the cost-effectiveness of scIgRT and ivIgRT suggests that significant cost savings could be made with a subcutaneous formulation as opposed to an intravenous preparation (53). A Canadian analysis indicated that every 37 patients treated with scIgRT in preference to ivIgRT resulted in sufficient financial savings to fund one nursing full-time equivalent (54).

Another approach to maximise cost-effectiveness is to de-escalate or withdraw IgRT when this approach is no longer necessary. Current guidelines do not provide clear advice with respect to the timing and indications for safe IgRT discontinuation. Therefore, in the absence of evidence-based recommendations for IgRT discontinuation clinical expertise and medical judgment should guide decision making. A reasonable approach could be a gradual IgRT discontinuation at one-year in patients with history of underlying disease in remission, a normal IgG trough level and evidence of immune reconstitution if CLL-directed therapy has been deployed. Following discontinuation, the criteria for the recommencement of IgRT should be the same as those for the primary indication.

There is a need therefore to develop a clinical trial wherein the efficacy, safety of IgRT replacement is compared with less costly prophylactic oral antibiotics in persons with CLL with secondary hypogammaglobulinemia.

## IgRT in CLL, beyond infection prophylaxis

IgRT is administered at distinct dosage in two different clinical contexts:- immunodeficient patients are treated with replacement doses of immunoglobulin, typically 0.2-0.4g/kilogram every 3 to 4 weeks whilst patients with autoimmune and inflammatory diseases are administered with very high doses of IgRT such as 0.5-2g/kilogram often over a single, or several days (39). In CLL, IgRT is generally believed to replace the missing antibodies and thereby prevent recurrent infections. However, the most important question remains: does IgRT mediate more complex mechanisms of immune reconstitution possibly with an associated with a direct anti-tumour effect rather than just preventing infections in CLL?

Spaner et al. showed that scIgRT preparations have the ability to impair BCR signalling, activation, and cytokine secretion by CLL-cells when stimulated *in vitro* by BCR ligation (9). This IgRT regimen decreased TNF-α and β2-microglobulin level (a marker of lymphocyte turnover), whilst a more intensive IgRT regimen decreased BCR activation. These findings could indicate that rather than acting solely as a replacement therapy, administered immunoglobulin may have activity against CLL-cells *in-vivo*. As immunoglobulin isotypes IgA and IgM may also be suppressed in CLL, the isotype content of immunoglobulin preparations could have different effects. Colado and colleagues compared effects of the pentaglobin (IVIgGMA) preparation which includes all immunoglobulin isotypes, versus standard preparations which contained only IgG, on leukemic and T-cells obtained from CLL patients (55). This study found that CLL-cell BCR signalling was reduced by both preparations. Whilst the addition of IVIgGMA to *in-vitro* cultures decreased venetoclax-induced T-cell apoptosis, this did not affect CLL-cell apoptosis in response to this drug. This may indicate an intrinsic CLL-cell resistance to apoptosis in the presence of IgRT and is perhaps supportive role of BTKi therapy as opposed to BCL-2 inhibition in CLL patients treated with IgRT.

Colado et al. also showed that the proliferation of T-cells from persons with CLL in response to TCR-stimulation or IL-15 was differentially affected by two different immunoglobulin preparations (55). There was enhanced inhibition of T-cell proliferation in response to anti-CD3 and IL-15 with intravenous IgG compared with IVIgGMA preparations. Moreover, IgM-enriched immunoglobulin preparations showed no benefits when used for the treatment of haematological patients with sepsis. This would suggest that polyclonal IgG based immunoglobulin preparations have more protective and immunomodulatory actions than enriched preparations.

## Conclusions

In conclusion, in the era of targeted therapy, IgRT should be offered to CLL patients as suggested by current guidelines (2). However such guidance can be criticised as the evidence is mainly derived from historic studies where CLL-directed treatment was predominantly with chemoimmunotherapy as opposed to the targeted agents currently in widespread use. There is little evidence in relation to the role of IgRT in persons with CLL treated with BTKi or inhibitors of BCL-2. The results of a recent, large, retrospective study suggests that adherence to IgRT guidelines positively correlates with fewer and less severe infections (6). This finding corroborates the results of a recent European expert conference on recommendations for the diagnosis and treatment of antibody deficiency with IgRT in patients with malignant hematologic diseases (56) This consensus document supports the initiation of IgRT in persons with IgG of less than 4g/L and at least one major infection treated with parenteral antibiotics or three infections over a twelve month period.

Prospective studies to assess the cost-effectiveness of IgRT and the impact on quality of life for persons with CLL are required. Furthermore, long-term IgRT for persons with CLL may not be necessary therefore a systematic approach to IgRT discontinuation is needed. Expert opinion suggests that IgRT could be discontinued after six months without an infection in the presence of evidence of immune reconstitution.

Finally, the successful navigation of the complex TME in CLL and the development of an understanding of the disease- and treatment-related immunosuppression is a significant challenge, but the development of BTKi has permitted fascinating insights into immune escape mechanisms present in CLL patients. We suggest that a mechanism of immunomodulation with a combination of B-cell-directed therapies and polyclonal immunoglobulin in high-risk CLL is possible and worthy of future investigation. Such combined modality immunomodulation could represent a novel therapeutic approach for CLL treatment.

## Author contributions

SK conceived the idea and wrote the main body of the paper. DA and SM directed, reviewed, and revised the manuscript. All authors contributed to the article and approved the submitted version.
